# Effects of left ventro-dorsal stream stimulation on novel tool use

**DOI:** 10.1093/cercor/bhag035

**Published:** 2026-04-07

**Authors:** Clara Seifert, Philipp Gulde, Thabea Kampe, Afra Wohlschläger, Joachim Hermsdörfer

**Affiliations:** Department Sport and Health Sciences, TUM School of Medicine and Health, Technical University of Munich, Am Olympiacampus 11, 80809 Munich, Germany; Department Sport and Health Sciences, TUM School of Medicine and Health, Technical University of Munich, Am Olympiacampus 11, 80809 Munich, Germany; Department Sport and Health Sciences, TUM School of Medicine and Health, Technical University of Munich, Am Olympiacampus 11, 80809 Munich, Germany; TUM-NIC, Department of Neuroradiology, TUM School of Medicine and Health, Technical University of Munich, Ismaningerstr. 22, 81675 Munich, Germany; Department Sport and Health Sciences, TUM School of Medicine and Health, Technical University of Munich, Am Olympiacampus 11, 80809 Munich, Germany

**Keywords:** kinematics, mechanical problem solving, novel tool use, TMS, ventro-dorsal stream

## Abstract

Mechanical problem solving enables using novel tools without primarily recalling semantic knowledge. In our previous fMRI study, the left ventro-dorsal stream, with clusters in the anterior supramarginal gyrus and ventral precentral gyrus, was active as healthy participants used novel tools. By using transcranial magnetic stimulation, the current study investigates the regions’ causal contributions. Participants were stimulated in the left anterior supramarginal gyrus or ventral precentral gyrus while planning to use novel tools to lift a cylinder. Each participant completed the tasks once under verum and once under sham stimulation. Performance accuracy and hand kinematics were assessed. Overall task performance remained unaffected by the stimulation at either site. However, stimulating the anterior supramarginal gyrus during early planning processes tended to reveal longer reaching paths and reduced velocity magnitudes when preparing the tool’s attachment. Stimulating the ventral precentral gyrus during later planning stages resulted in delayed movement initiation and extended deceleration as the tool approaches and lifts the cylinder. Kinematic compensation behavior may allow successful task performance despite the stimulation. The need for compensation may arise from subtle challenges in decision-making and tool-cylinder-matching processes, supported by the anterior supramarginal gyrus during early processing stages. A more detailed analysis of the tool-cylinder match may be needed when later processes supported by the ventral precentral gyrus are disrupted.

## Introduction

### Novel tool use and mechanical problem solving

Using tools represents an important ability in everyday life, and observations of impaired tool-use performance in neurological patients with apraxia initiated the exploration of the underlying neural mechanisms in greater detail. Although in many everyday life situations, familiar tools are used to achieve a goal, humans are also capable of handling unknown, thus novel, tools. While in these situations, little reliance on previously gained tool-related semantic knowledge is possible, solutions can be generated based on an individual’s mechanical problem solving ability (Goldenberg and Hagmann 1998; Osiurak et al. 2009). Instead of predominantly retrieving tool-related semantic knowledge, individuals are able to process visual and tactile features of an object, while considering basic mechanical and physical principles (Goldenberg and Hagmann 1998). This procedure enables one to understand the proper usage of an unknown tool with little reliance on prior knowledge and accessible instructions. Mechanical problem solving helps healthy individuals to use novel tools and apply known tools in an unfamiliar context (Goldenberg and Spatt 2009), and allows patients with limited access to tool-related knowledge compensating to manipulate and handle tools (Hodges et al. 2000). While past studies provided evidence for the different contributions of a network comprising the left-hemispheric ventral, ventro-dorsal, and dorso-dorsal streams in familiar tool-use tasks (Binkofski and Buxbaum 2013; Brandi et al. 2014; Metaireau et al. 2024), our previous study looked at the brain network specifically recruited, when healthy participants are using novel, unknown tools (Seifert et al. 2025). In this study, participants conducted tasks from an MRI-compatible, extended version of a test developed for the assessment of novel tool use in apraxia (Goldenberg and Hagmann 1998; Randerath et al. 2017) inside a scanner. Each trial of this test involved a unique combination of a completely unknown tool and an object that had to be lifted. Although some tasks involving these novel tools may have offered participants the opportunity to recall tool-related semantic knowledge and to draw analogies between the current problem and existing knowledge (eg regarding the hand shape needed for grasping), it is assumed that they primarily relied on their mechanical problem solving skills to solve the task. This assumption is further supported by the findings from our previous fMRI study, and the lack of the ventral stream contribution compared to tasks involving familiar tools, suggesting that semantic knowledge may have played a subordinate role in this context (Seifert et al. 2025). Although bilateral contribution of the inferior parietal lobe has been reported in past studies (McDowell et al. 2018; Styrkowiec et al. 2019; Ras et al. 2022), the results obtained in our previous study indicate a predominant activation in the left hemisphere (Seifert et al. 2025), in line with the majority of previous studies investigating novel (Weisberg et al. 2007; Menz et al. 2010; Seidel et al. 2023) as well as familiar tool use (Brandi et al. 2014). Interestingly, this activation was only distinct from an active baseline condition during the planning phase of these tasks and similar brain activation patterns showed up, independent of whether participants had to plan to point or to actually grasp and lift the cylinder. This network mainly covers the left-hemispheric ventro-dorsal stream, which extends from the medial superior temporal areas through the inferior parietal lobe and continues anteriorly to the ventral premotor cortex (Rizzolatti and Matelli 2003). Two clusters within this stream were particularly active: one covering the left anterior part of the supramarginal gyrus (aSMG) and the other covering the left ventral subdivision of the precentral gyrus (vPreCG). This fMRI study provided new insights into brain areas involved when healthy participants perform tasks with real novel tools that required mechanical problem solving. However, a gap of knowledge remains to understand how the regions within this activated network causally contribute to successful task performance.

### Studying the causality of the left ventro-dorsal stream

While this past fMRI study allowed visualizing the whole-brain network involved in mechanical problem solving tasks, the resulting fMRI data do not allow to conclude a causal influence of the activated brain regions on performing (steps of) these tasks properly (Pascual-Leone et al. 1999; Logothetis 2008; Ruff et al. 2009). Therefore, our previous study raised the question of how the left aSMG and vPreCG (as part of the left ventro-dorsal stream) contribute to performing these tasks including novel tools successfully. Understanding how this stream causally contributes to tool-related task performance is primarily based on observations of patients with ventro-dorsal lesions and data obtained from animal studies (Rizzolatti and Matelli 2003).

Several lesion studies involving apraxic patients have confirmed the importance of the ventro-dorsal stream for successful manipulation of novel tools (eg Goldenberg and Hagmann 1998; Goldenberg and Spatt 2009; Stoll et al. 2022; Rounis and Binkofski 2023). Successful action organization, including action understanding and spatial perception, is proposed to depend on the functionality of the ventro-dorsal stream (Rizzolatti and Matelli 2003). Additionally, the stream represents a “use” system for object-related actions (Binkofski and Buxbaum 2013) and is involved in processing intrinsic object properties and stable affordances. Moreover, damages to the left ventro-dorsal stream can be associated with impaired affordance judgments. Individuals may rely on more flexible rather than stable criteria when judging eg about the reachability of an object (Randerath et al. 2021). Additionally, they may struggle to integrate body and object properties into action plans (Buxbaum and Randerath 2018). Rounis and Binkofski (2023) summarize the current evidence and propose that left ventro-dorsal stream damage perturbs the successful inference of an object’s function by processing its structure, as it was similarly described by Goldenberg and Hagmann (1998) and linked to impaired mechanical problem solving strategies.

These insights, primarily influenced by observations of deficits in patients with apraxia, have shed light on the causal role of the ventro-dorsal stream in performing tool-related tasks. However, little is known about the causal relationship between the recruited areas within the left ventro-dorsal stream and the task performance required to solve mechanical problems in healthy individuals. Thus, implementing a “virtual lesion” protocol through the use of transcranial magnetic stimulation (TMS) may offer an effective way to overcome these limitations by allowing to test the causal contributions of specific brain areas in healthy participants to successful task performance (Pascual-Leone et al. 1999). Thus, if one brain region is indeed responsible for proper task execution in healthy individuals, its stimulation should result in observable behavioral deficits. Therefore, the current study builds on the findings regarding the identified network (Seifert et al. 2025) by investigating the causal contributions of activated brain regions to the observed behavioral outcomes.

### TMS studies and tool use in healthy participants

The causal role of the inferior parietal lobe and specifically the SMG, as part of the ventro-dorsal stream, has been examined in the context of reaching and grasping movements in healthy participants (Tunik et al. 2005; Koch et al. 2010; Potok et al. 2019). Changes in grasp orientation and grasp aperture following stimulation of the anterior intraparietal sulcus (aIPS) suggest its role in error-detection processes (Tunik et al. 2005). In contrast, alterations in cup-grasping were observed after stimulating the SMG (Koch et al. 2010). Additionally, stimulation of the angular gyrus indicates its involvement in processing object positions (Koch et al. 2010). Research has expanded beyond the role of the inferior parietal lobe in simple reach-to-grasp movements to investigate its specific contribution to more complex tasks, including tool use. For instance, one study by Andres et al. (2017) found that stimulating the left SMG led to prolonged response latencies in a task requesting to process finger positioning for object use. Similarly, Pelgrims et al. (2011) demonstrated longer reaction times when stimulating the left SMG in a task requiring participants to evaluate the hand posture for proper tool use. Additionally, Ishibashi et al. (2011) highlighted distinct roles for the left anterior temporal and the left inferior parietal lobes, as their stimulation resulted in different types of deficits. Their findings suggest that the inferior parietal lobe is relevant for tool manipulation, while the anterior temporal lobe contributes to proper rating of a tool’s function (Ishibashi et al. 2011). Another TMS study examined the specific temporal role of the SMG while healthy individuals plan functional grasps of tools, highlighting its involvement in early planning processes probably needed to integrate relevant structural and functional features of tools (Potok et al. 2019).

These studies, involving tool-related tasks, typically utilized experimental stimuli presented visually on a screen, requiring participants to judge finger positions (Andres et al. 2017), plan functional grasps (Potok et al. 2019), assess tool function or object manipulation (Ishibashi et al. 2011), and evaluate context and hand positions in tool use (Pelgrims et al. 2011) or pantomime tasks (Kleineberg et al. 2022).

Many of these studies focused on the effects of TMS on reaction times, response latencies, or overall performance, mainly referring to the number of committed errors. However, some aimed to explore the movement profiles of tool-related actions in greater detail. This approach includes precise tracking of hand movements, offering a closer look at the kinematic profiles of the movements and allowing a more comprehensive understanding of abnormalities shown by apraxic patients. By investigating the kinematic movement characteristics, it became apparent that the velocity profile of patients with left-hemispheric damage was prolonged in time (Hermsdörfer et al. 1999), while other abnormalities were observed, regarding the movement amplitude (Hermsdörfer et al. 2012), the movement coordination (Schaefer et al. 2009; Mutha et al. 2010), the hand rotation (Hermsdörfer et al. 2012) as well as the movement speed (Schaefer et al. 2009; Hermsdörfer et al. 2013) and movement direction (Schaefer et al. 2009; Hermsdörfer et al. 2013).

In one of the studies using motion tracking of the hand while testing the effects of TMS in healthy participants, stimulating the left inferior frontal gyrus (pars opercularis) or the SMG revealed delayed reaction times when grasping to pour (goal) a cup (tool), without influencing the onset or offset of the movement, suggesting that these two areas play a specific role in planning goal-oriented actions (Tunik et al. 2008). Similarly, McDowell et al. (2018) investigated movement time and the time it took to reach the maximum velocity of hand movement while participants reached toward common tools known from everyday life. Bilateral stimulation of the SMG produced increased movement time and shorter time to reach the maximum velocity, being further associated with longer deceleration while the target tool was approached (McDowell et al. 2018).

To summarize the existing evidence regarding the causal contributions of brain regions involved in tool-related tasks, multiple studies have applied a “virtual lesion” protocol to mainly examine the role of the inferior parietal lobe. Most studies primarily focused on reaction times and overall performance as main outcomes, while only a few explored the effects of TMS on kinematic movement characteristics. Additionally, many of these studies elucidated reaching and grasping behavior to gain insights about the causal contributions of the stimulated areas. Experimental stimuli were presented either visually on a screen (eg Pelgrims et al. 2011; Andres et al. 2017; Potok et al. 2019) or as tangible items (McDowell et al. 2018). While these studies provided valuable insights into the specific contribution of the stimulated areas to tool-related task performance, evidence is lacking regarding the effects of TMS when healthy participants perform tasks using real tools. Thus, given our interest in using novel, unfamiliar tools, we applied TMS to two clusters of the network reported in our previous fMRI study (Seifert et al. 2025). We hypothesized that worse task performance would be observed in trials with verum compared to sham stimulation. Besides analyzing the number of committed errors, we tracked the movement of the left hand to further investigate the effects of TMS on the kinematic profile. Assuming the verum stimulation producing a virtual lesion in healthy individuals, we expected disturbed kinematic profiles for trials receiving verum stimulation. In addition, since TMS provides the opportunity to gain specific insights into the temporal contributions of brain regions during planning tool-related tasks, as demonstrated in previous studies (Tunik et al. 2005; Potok et al. 2019), we were interested in the effects of the stimulation applied during early or late planning phases. In correspondence with the assessment of the left hand in clinical apraxia testing and our approach in the fMRI study on novel tool use, we investigated the left hand ipsilateral to the stimulation in the left ventro-dorsal stream.

Considering the role of the left inferior parietal lobe (IPL) acting as a functional core region for tool use within the ventro-dorsal stream (Randerath 2023), stimulation to the aSMG, an important subregion, is expected to lead to comprehensive deficits of the cognitive abilities needed for accurately planning solutions to mechanical problems. The left vPreCG turned out as another important cluster contributing to novel tool use in our previous fMRI study, and it is located in the ventral premotor cortex, which is part of the motor networks that support movement control (Kantak et al. 2012). Considering that the aspects of planning tool-use movements are not hand specific, we expected to observe deficits in kinematic features such as movement velocity and movement smoothness following stimulation of this area in the hemisphere.

## Methods

### Participants

In total, 42 participants were recruited. One participant quit the study due to uncomfortable feelings, and another participant was excluded because of technical issues with the determination of the resting motor threshold (rMT). Data from three other persons did not enter the data analysis because of failed camera calibration. Thus, a final sample of 37 participants was included in the current analysis. Each participant was randomly assigned to one of two stimulation sites, determined by the group-level peak coordinates from the previous fMRI study. All of them fulfilled inclusion criteria for TMS (eg no history of seizures, no history of neurological or psychiatric diseases) and MRI (eg claustrophobia) as a structural brain scan was acquired first. Only right-handed individuals were included in the study, with their handedness verified by the Edinburgh Handedness Inventory (Oldfield 1971), requiring a laterality quotient of $\ge 75$. Each participant provided written informed consent in alignment with the principles of the Declaration of Helsinki. The study protocol received approval from the ethics committee (618/20S-KH) of the School of Medicine and Health at the Technical University of Munich.

### Stimuli and task

The experimental stimuli used in the current TMS study were identical to those employed in our previous fMRI study (Seifert et al. 2025). As we stimulated spots which were identified in our previous fMRI study (left aSMG and left vPreCG, see below), we decided to use the same Tool Carousel, containing the MRI-compatible stimuli from the Novel Tool Test (Goldenberg and Hagmann 1998; Randerath et al. 2017), outside the scanner. This carousel consists of four 90° segments. Each segment featured three tools and one cylinder. A total of 14 unique tool-cylinder combinations were available. To avoid upcoming biases due to a specific sequence of stimulus presentation, participants were pseudo-randomly assigned to one out of four distinct versions, each characterized by a unique order in which tool-cylinder combinations were presented. An overview of the stimulus material is presented in the supplementary material (Figure S2).

Participants were seated in front of a table, and the Tool Carousel was situated 30 cm away from the table’s edge, ensuring that participants could comfortably access the contained tools. In addition to the experimental setup, participants were provided with shutter glasses (PLATO; Translucent Technologies, Inc., Toronto, Canada). The entire experiment was structured into two runs, both comprising an identical sequence of presented tool-cylinder combinations. Each run consisted of 14 trials, covering all available 14 tool-cylinder combinations. These runs were further divided into two parts, with the key distinction being the type of coil used. Participants were blinded and randomly assigned to one of two conditions, determining whether either the verum or the sham stimulation was applied to the first or last seven presented tool-cylinder combinations of one run. Participants in group A underwent the following sequence: They initially received verum stimulation in the first part (seven tool-cylinder combinations) of the first run, followed by sham stimulation in the second part of the same run. The second run was again separated into two parts, starting with sham stimulation in the first part and concluding with verum stimulation in the second part. Verum and sham stimulation was switched in group B. In accordance with this protocol, each participant performed each tool-cylinder task twice: once under the influence of verum stimulation and once under the influence of sham stimulation.

**Figure 1:**
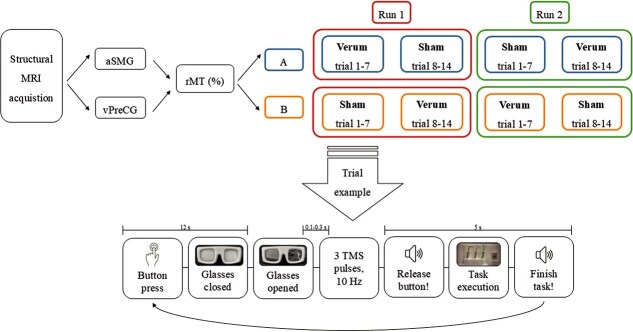
Visualization of the experimental procedure. *Note.* The experimental procedure included randomized assignment of participants to one of the two stimulation sites and the condition of the stimulation type order (A or B). One trial was characterized by the following time course: button press (0s); glasses close (12s); glasses open; TMS pulses; start signal to select the most suitable tool and lift the cylinder out of the socket (5s); re-pressing the button.

A detailed overview of the study design, including randomized assignments of participants, is presented in Fig. 1.

In order to ensure comparability with data from persons with apraxia following a stroke, who often show right-sided hemiparesis, and to avoid motor confounding due to stimulation of left-hemispheric areas, participants were asked to perform all tasks exclusively with their left hand.

In each trial, participants then underwent the following procedure: They initiated the trial by continuously pressing a button on the table in front of them. This button press triggered the goggles they were wearing to close. After ~12s, the goggles reopened. Subsequently, three TMS pulses with a frequency of 10 Hz were delivered. The onset of the first released pulse was falling within a randomly varying, continuous interval ranging from 100 to 300ms after the goggles had reopened and the planning phase started. This random variation minimized the risk for potential anticipation and conditioning effects. Immediately following the delivery of the last TMS pulse, a sound signaled to the participants that they could release the button and begin the task (see trial example in Fig. 1).

**Figure 2:**
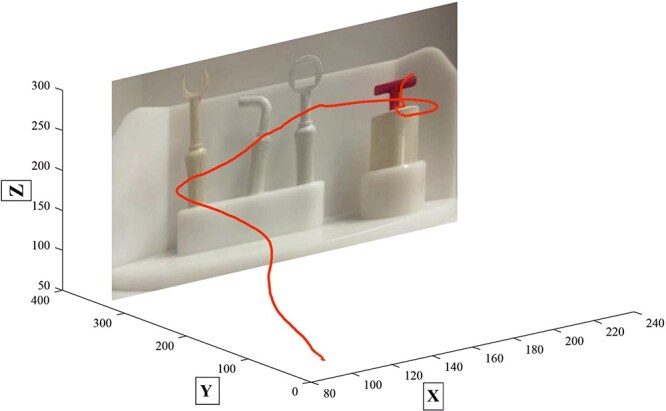
Exemplary trial depicting three novel tools and one cylinder with the trajectory profile overlaid. *Note.* The red line illustrates an exemplary trajectory profile, starting from the starting point, moving toward the tool that was grasped, and moved and used to lift the cylinder; inserted picture is not to scale.

The task involved selecting a tool with an appropriate key part capable of lifting the cylinder within the presented segment. An example trial is depicted in Fig. 2 with an overlaid trajectory profile. Participants had a 5-s window to complete this task. After the 5s, the same sound appeared, indicating that participants should return the cylinder and the used tool and then press the button again. Upon pressing the button again, the goggles closed, and the trial concluded. Between the closing and reopening of the goggles for the next trial, the Tool Carousel was rotated and the cylinder as well as the according tools were replaced and prepared for the following trial.

### TMS procedure

To deliver brain stimulation in our study, we employed the TMS device PowerMAG 100 Research (Mag & More, Munich). This device is equipped with a figure-of-eight-shaped coil (Double coil PMD70-pCool), characterized by a pulse duration of 160 $\mathrm{\mu}$s and a maximum field intensity of 2 Tesla. Our experiment included both verum and sham stimulation, the latter achieved using a sham coil (Double coil PMD70-pCool-SHAM). Notably, both coils shared identical visual, tactile, and auditory characteristics to ensure a consistent experience for participants.

To establish each individual’s resting motor threshold (rMT), we assessed the motor-evoked potential of the right first dorsal interosseous (FDI) muscle. This assessment was conducted by applying single-pulse transcranial magnetic stimulation over the left primary motor cortex (M1), while simultaneously recording the muscle response via electromyography (LabChart; ADInstruments). While determining an individual’s rMT, we positioned the verum coil contralateral to the target muscle (right first dorsal interosseous muscle). The coil was centered over the C3 hand area, specifically 3 to 4 cm lateral and 1 to 2 cm anterior to the vertex. To ensure precise placement, the coil grip was inclined in a postero-lateral direction at a 45° angle to the sagittal axis. This positioning served as our starting point to identify the exact location of maximum excitability for the muscle. The rMT was set to the stimulation intensity required to elicit 8 out of 10 motor-evoked potentials in the right FDI muscle.

Once an individual’s rMT was determined, we proceeded to apply a magnetic field intensity set at 120% of this threshold for the actual experiment, similar to the procedure described by Ishibashi et al. (2011). This approach revealed an average stimulation intensity across all participants of *M* = 60.8% (*SD* = 8.1). A three-pulse stimulation protocol characterized by a frequency of 10 Hz was applied. The onset of stimulation varied randomly within a continuous range of 100 to 300ms following the participants’ initial exposure to the presented carousel segment containing one specific tool-cylinder combination. The first moment participants had access to the task represented the beginning of the planning phase. The varied stimulation onsets after the beginning of the planning phase were used to decrease an individual’s anticipation ability, thereby minimizing potential risks of conditioning effects.

**Figure 3:**
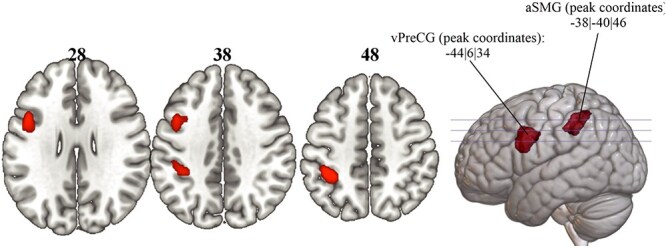
Depicting the results of the previous fMRI study, revealing the two stimulation sites: aSMG and vPreCG.

Two specific target locations were identified based on the findings of our previous fMRI study (Seifert et al. 2025). As we identified a similar network for both tool selection and tool use tasks, we decided to merge the tool selection and tool use tasks into a single combined task, in accordance to the clinical assessment (Goldenberg and Hagmann 1998). This procedure has the advantage of minimizing the chances of interacting with the same tool-cylinder combinations and to ensure the novel characteristic of the (limited) amount of tool-cylinder combinations available. Thus, a conjunction analysis of the relevant fMRI second-level contrasts (tool selection and tool use tasks contrasted against their respective active baseline conditions during the planning phase for just the left hand used) revealed two key regions belonging to the ventro-dorsal stream (Seifert et al. 2025), as depicted in Fig. 3: One cluster contains the left aSMG (peak coordinates in MNI space: −38$\mid$−40$\mid$46) and the other cluster comprises the ventral part of the left precentral gyrus (vPreCG) (MNI peak coordinates: −44$\mid$6$\mid$34). These specified peak coordinates were used as the two stimulation sites for all participants who did not take part in the previous fMRI study. Participants were randomly assigned to one of these stimulation sites. The appropriate position of the verum and the sham coil was enabled due to neuronavigation software (PowerMAG View!; MAG and More, Munich), with individual high-resolution T1 anatomical brain scans being acquired first. For a minority of four individuals who had participated in the previous fMRI study, their individual peak coordinates within one of these two clusters were used as the stimulation site (see “Individualized target determination” section).

### Individualized target determination

For four participants, individualized target determination was possible, as they took part in the previous fMRI as well as in the current TMS study. First, a combined contrast (tool use and tool selection) was calculated for the planning phases of trials performed with the left hand at a first level. This contrast was then masked using the second-level group analysis results to reveal the individual peak coordinates in the aSMG or vPreCG cluster. Furthermore, the resulting contrast was transformed into native space using the inverse deformation and the peak coordinates served as the following target stimulation (further details are listed in the supplementary material Table S7). Due to the fact that >1.5 years passed between participation in the fMRI study and the subsequent TMS study, we assume that the requirements and processes for completing the task are similar to those of the other, newly recruited, participants. This assumption is also further supported by the behavioral data.

### Video analysis and motion capture

**Figure 4:**
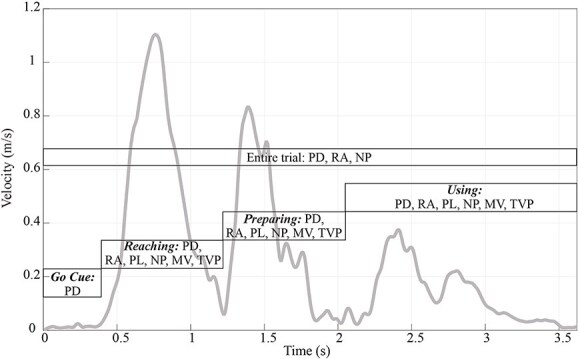
Exemplary smoothed velocity profile. *Note.* Kinematic parameters were calculated for different phases; PD = phase duration, RA = relative activity, PL = pathlength, NP = number of velocity peaks per meter, MV = maximum velocity, and TVP = timepoint of maximum velocity relative to the phase duration.

A three-dimensional motion-capturing system (Qualisys Inc., Gothenburg, Sweden) was used to record positional data of participants’ left-hand movements during task execution. These positional data were gathered at 100 Hz using three cameras (Oqus, 3MP resolution). An additional camera was employed for the purpose of video recordings. These video recordings enabled the evaluation of performance measures, including the assessments of task-related errors committed by the participants, as well as the identification of the different phases during the entire task execution. The positional data of the marker attached to the base of the left hand’s index finger were labeled using the QTM software (Qualisys Track Manager) and used for further kinematic parameter calculations in MATLAB (MATLAB R2025a; MathWorks MA). To address missing frames, a polynomial interpolation method was applied. The Euclidean distance of the positional data, being used to calculate the hand movement’s velocity profile, was further smoothed using a 0.42-s “loess” filter (local regression) (Gulde et al. 2018). An exemplary velocity profile is illustrated in Fig. 4. One entire trial lasted from the appearance of the signal to release the button until the task was completed, thus when the cylinder was lifted out of the socket. The velocity profile for an entire trial was divided into distinct phases based on video frames. The *Go Cue* phase included the period from when the signal appeared until the button was released and ended one frame before the hand movement began. The *Reaching* phase refers to the time starting from the first video frame in which hand movement was detected until one frame before the fingers contacted the tool handle. The *Preparing* phase began when the first frame showed the grasped tool and lasted until the tool was moved toward the cylinder. Finally, the *Using* phase started at the first frame when the tool contacted the cylinder and continued until the cylinder was lifted out of its socket. This method ensured that each video frame was assigned to one specific phase. Kinematic parameters were then calculated for each phase to provide a comprehensive understanding of the time before the movement, its onset, and the movement itself. A more detailed overview of these phases is available in the supplementary material (Figure S1).

Based on theoretical assumptions, observations of participants performing the tasks and experience from previous kinematic testing (Hermsdörfer et al. 2006, 2013), we were particularly interested in the following six kinematic parameters:

1. PD (phase duration): time taken per phase (s)
2. RA (relative activity): time hand was moving faster than 0.05 m/s relative to duration, higher values indicating a more fluent, less interrupted execution (%)
3. PL (pathlength): length of the hand’s trajectory in three-dimensional space, higher values indicating more work, ie less efficiency of execution (m)
4. NP (number of velocity peaks per meter): velocity peaks (with a minimum peak prominence 0.05 m/s) per meter, representing movement smoothness with higher values depicting less fluid movements (1 peak/m)
5. MV (maximum velocity): maximum of the velocity profile (m/s)
6. TVP (timepoint of velocity peak): timepoint of the maximum velocity relative to phase duration, higher values depicting later peaks (%)

All parameter calculations are based on the Euclidean distance and were reported in previous studies on kinematic differences of patients in clinical testing (de los Reyes-Guzmán et al. 2014) and activities of daily living (Gulde and Hermsdörfer 2017; Gulde et al. 2018). The time taken by participants to start moving their hand (*Go Cue*) and to complete one entire trial was measured, as well as the durations of the *Reaching*, *Preparing*, and *Using* phases separately. Additionally, the maximum velocity achieved within each of these three phases and the timing of this peak velocity were calculated.

Furthermore, the length of the trajectory traveled in three-dimensional space was determined for each of the three subphases. Also, the number of detected velocity peaks, representing a measure of movement smoothness (continuous, uninterrupted movements; Gulde and Hermsdörfer 2018), and the relative time spent active throughout the entire trial as well as specifically during the *Reaching*, *Preparing*, and *Using* phases were calculated.

### Evaluation of video recordings

The video-recorded data were used following two purposes: to separate the entire trial into the specific phases described earlier and to evaluate participants’ performance. The same evaluation scheme as in the previous fMRI study was applied (Seifert et al. 2025). For the selection score, either 0 or 1 point was awarded. In case the proper tool was selected (including one self-correction), 1 point was awarded. In addition, participants also received 1 point in case the wrong tool was selected, but nevertheless, the final goal (lifting the cylinder out of the socket) was properly achieved. The production was evaluated with scores ranging from 0 to 3. Three points were awarded for complete task execution, 2 points for grasping the tool, moving it to the cylinder without attaching it, 1 point for grasping the tool but not moving it to the cylinder, and 0 points if the participants did not do anything after they received the signal to start the task execution.

### Statistical analysis

The statistical analysis for both the video-recorded data and the positional data gained from motion tracking was conducted using RStudio 4.5.0 (Posit. 2025). Each participant performed the 14 available tool-cylinder combinations twice—once under verum stimulation and once under sham stimulation. Some trials did not enter the final statistical analysis due to missing video data, hand movements occurring before the starting signal, or errors in the presentation of the experimental stimuli. This left an average of 13.5 valid trials conducted per participant for verum and 13.1 for sham stimulation. The $\alpha$-level was set to 0.05.

#### Analysis of video recordings

For the analysis of the video data, average scores for both selection and production were calculated for each participant and for each type of stimulation they received (verum and sham). Two separate ANOVAs were further performed: one to assess the main and the interaction effects of stimulation location, stimulation type, and timepoint on selection scores; and the other to evaluate their impact on production scores. The evaluation of the video data was carried out by two independent researchers using the same evaluation criteria. Cohen’s kappa yielded an acceptable inter-rater reliability of $\kappa =0.95$, 95% CI [0.92, 0.97] for selection and $\kappa =0.82$, 95% CI [0.79, 0.86] for production scores.

#### Analysis of positional data

After preprocessing the positional data, the resulting kinematic parameters were used for further statistical analyses. We combined the kinematic parameters measured during each phase to build logistic regression models that accurately classify the characteristics of the stimulation, including the type and site. The model that solely included trials conducted under sham stimulation demonstrated a strong ability to distinguish between the two stimulation sites, aSMG and vPreCG, achieving an area under the curve (AUC) of 0.85 (95% CI: [0.82 to 0.89]). This indicates that the kinematic data gathered from sham-stimulated trials are influenced by the specific site being stimulated. This observation is discussed further below and informed the decision to create two separate logistic regression models for each stimulation site. Thus, we aimed to create two models, one for each stimulation site, that can differentiate between verum and sham stimulation for trials performed under either stimulation to the aSMG or to the vPreCG. All potential predictor variables were scaled. To gain insights into the temporal contributions of the two selected stimulation sites, we included the stimulation timepoint to build interaction terms with the kinematic predictors. To prevent model-specific cutoff scores for the stimulation timepoint, we binarized it by a median split, resulting in a cutoff score of 0.182s. Thus, trials in which the first pulse occurred before 0.182s were classified as “early-stimulated,” while trials with the onset of stimulation after this threshold were classified as “late-stimulated,” independent of the stimulation site. For the odds ratio (OR) of the stimulation timepoint (early/late), “early” served as the reference category. For the OR of the stimulation type (sham/verum), “sham” served as the reference category. Initially, we specified a full model with predictors including the 22 kinematic parameters depicted for the different phases in Fig. 4, the selection and production scores as well as the categorized timepoints of stimulation (early/late). Moreover, interactions of all kinematic parameters with the binarized timepoint of stimulation were included in the model as potential predictors. The models were refined by a backward selection procedure, with a critical variance inflation factor (VIF) of 5 and an $\alpha$-level of 0.10. This procedure ensured minimization of artificial variance and overfitting. A detailed overview of the applied model selection procedure is provided in the supplementary material (see Table S1–S6). In addition, correlation matrices including all kinematic predictors for both models is available in Figure S3 and Figure S4 of the supplementary material.

Receiver operating characteristic (ROC) curves were further generated, and their respective AUC was calculated to evaluate the predictive performance of the models. Moreover, both logistic regression models were compared against a Null-model, without any predictors included. Model comparison was done by conducting likelihood-ratio tests (LRT).

## Results

### Participant characteristics

Participant characteristics, including age, sex, handedness (Oldfield 1971), and the intensity of stimulation, are depicted in Table 1. These characteristics were compared between the groups.

**Table 1:** Participant characteristics by stimulation site.

	Left aSMG	Left vPreCG	*P*-value
*n*	18	19	
Female (%)	55.6	57.9	0.998
Age (years), *M* (*SD*)	26.1 (5.77)	25.8 (4.44)	0.851
EHI score, *M*	89.7	91.1	0.665
Stimulation intensity (%), *M* (*SD*)	62.1 (5.95)	59.4 (9.67)	0.311

*Note.* EHI = Edinburgh Handedness Inventory (Oldfield 1971). Stimulation intensity represents 120% of the identified rMT (intensity needed to elicit 8 out of 10 motor-evoked potentials); *P*-values were obtained by $\chi$^2^ and independent samples *t*-tests.

### Performance

**Figure 5:**
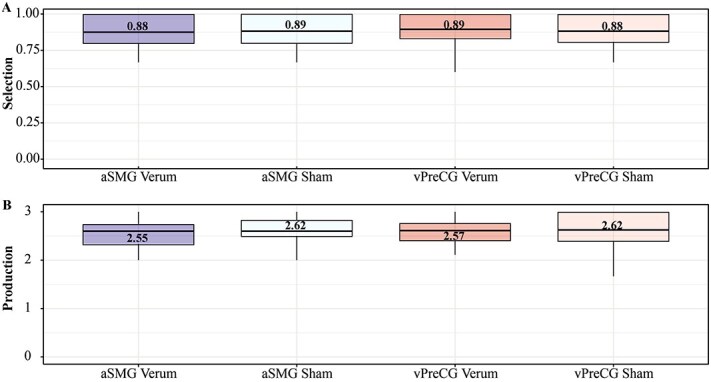
Performance scores. *Note.* Depiction of the averaged performance scores including both stimulation sites and stimulation types for selection (A) and production (B). Selection scores were either 0 or 1, while production scores ranged from 0 to 3.

In general, the participants performed without clear errors in the task. Figure 5 shows boxplots of the averaged scores for the four experimental conditions. For both stimulation types (verum/sham) across the two stimulation sites (aSMG/vPreCG), similar selection scores were achieved, with averages between 0.88 and 0.89. Likewise, the averaged production scores demonstrated only small variations between the stimulation types for both stimulation sites, ranging from 2.55 to 2.62. The results of the calculated ANOVAs indicate that the selection scores were neither influenced by the type of stimulation alone ($F\left(1,35\right)=0.03,P=0.863,\eta{p}^2=0.001$) nor in interaction with the stimulation site ($F\left(1,35\right)=0.64,P=0.430,\eta{p}^2=0.018$). The timepoint of stimulation showed a significant main effect ($F\left(1,35\right)=4.32,P=0.045,\eta{p}^2=0.11$). Thus, selection scores were lower when the stimulation occurred early compared to late (*M*_Early_ = 0.86 $\pm 0.12$; *M*_Late_ = 0.91 $\pm 0.11$; *P* = 0.045). The interaction of the stimulation timepoint with the stimulation type ($F\left(1,35\right)=0.06,P=0.81,\eta{p}^2=0.002$) or the stimulation site ($F\left(1,35\right)=0.48,P=0.494,\eta{p}^2=0.013$) was not significant. Similarly, neither the stimulation type ($F\left(1,35\right)=2.47,P=0.125,\eta{p}^2=0.07$) nor its interaction with the stimulation site ($F\left(1,35\right)=0.11,P=0.748,\eta{p}^2=0.003$) or timepoint of stimulation ($F\left(1,35\right)=0.07,P=0.789,\eta{p}^2=0.002$) showed any significant impact on the production scores.

### Positional data

**Figure 6:**
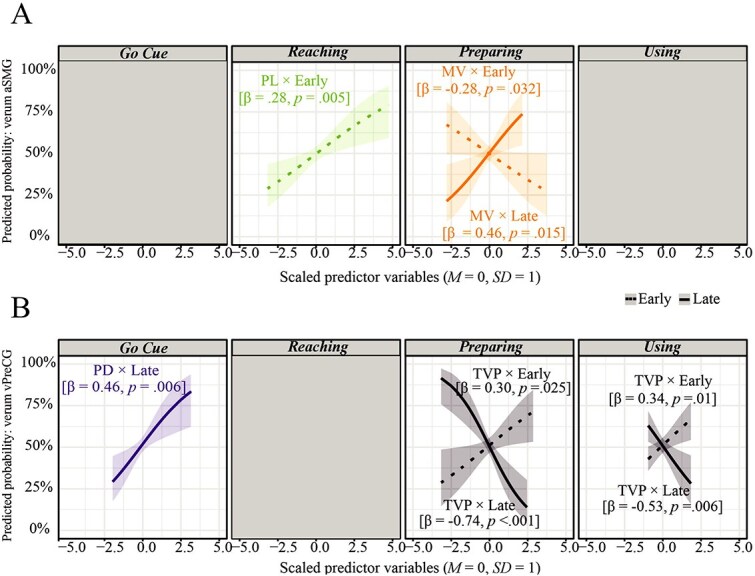
Predictive probabilities. *Note.* Predictive probabilities of significant parameters (*P* $<$ 0.05) predicting verum stimulation for aSMG (A) and vPreCG stimulated trials (B); kinematic parameters are scaled (*M* = 0, *SD* = 1); panels indicate the phase of the identified predictive parameter; PL = pathlength, PD = phase duration, TVP = timepoint of maximum velocity relative to the duration, and MV = maximum velocity; shaded bands represent 95% confidence intervals (CIs); dotted for early and solid lines for late-stimulated trials.

The final reduced model for predicting the stimulation type (verum/sham) within the aSMG group identified three significant predictors. The predictive probabilities for these significant predictors are shown in Fig. 6A. According to the selected reference level, the probability of classifying a trial performed under verum stimulation is depicted on the *y*-axis. Table 2 provides an overview of the significant predictors.

**Table 2:** Kinematic predictors interacting with the timepoint of stimulation to predict the stimulation type within the aSMG group (AUC = 0.60).

Phase	Parameter	β	*P*-value	VIF	*M*_Verum_	*M*_Sham_
*Reaching*	PL × early	0.28	0.005	1.07	0.43m ± 0.06m	0.40m ± 0.06m
*Preparing*	MV × early	−0.28	0.032	1.99	0.75 (m/s) ± 0.18 (m/s)	0.80 (m/s) ± 0.17 (m/s)
*Preparing*	MV × late	0.46	0.015	1.89	0.76 (m/s) ± 0.15 (m/s)	0.72 (m/s) ± 0.13 (m/s)

*Note.* PL = pathlength; MV = maximum velocity; *M* ± *SD*

Parameters from the *Reaching* and *Preparing* phases significantly contributed to distinguish between verum and sham-stimulated trials within the aSMG group. In trials in which pulses were delivered early, a longer pathlength (*Reaching* PL: $\beta$ = 0.28, *P* = 0.005, VIF = 1.07) while reaching toward the tools increased the probability to classify a trial as being performed under the influence of verum stimulation in the aSMG group. Moreover, the maximum velocity during the *Preparing* significantly predicted the type of stimulation. While during early stimulation a reduced maximum velocity (*Preparing* MV: $\beta$ = −0.28, *P* = 0.032, VIF = 1.99) increased the probability of a trial being performed under verum stimulation, the opposite holds true for late-stimulated trials, with higher velocity magnitudes (*Preparing* MV: $\beta$ = 0.46, *P* = 0.015, VIF = 1.89) increasing the probability of a trial being conducted under verum stimulation.

The final reduced model for predicting the type of stimulation within the vPreCG group identified five predictors. The predictive probabilities are shown in Fig. 6B. According to the selected reference level, the probability of classifying a trial as performed under verum stimulation is depicted on the *y*-axis. Table 3 provides an overview of the significant predictors.

**Table 3:** Kinematic predictors interacting with the timepoint of stimulation to predict the stimulation type within the vPreCG group (AUC = 0.62).

Phase	Parameter	β	*p*-value	VIF	*M* _Verum_	*M* _Sham_
*Go Cue*	PD × late	0.46	0.006	1.07	0.60s ± 0.25s	0.55s ± 0.22s
*Preparing*	TVP × early	0.30	0.025	2.02	53.4% ± 8.8%	50.9% ± 7.5%
*Preparing*	TVP × late	−0.74	<0.001	2.03	46.9% ± 8.5%	52.4% ± 6.9%
*Using*	TVP × early	0.34	0.01	1.99	42.0% ± 12.8%	32.5% ± 16.0%
*Using*	TVP × late	−0.53	0.006	1.93	32.1% ± 12.9%	33.7% ± 21.2%

*Note.* PD = phase duration; TVP = timepoint of maximum velocity; *M* ± *SD*

Parameters from the *Go Cue*, *Preparing*, and *Using* phases significantly contributed to distinguish between verum and sham-stimulated trials within the vPreCG group. Under late stimulation, the longer it took participants to start moving their hand toward the tool, the higher the probability of classifying a trial as being performed under verum stimulation (*Go Cue* PD: $\beta$ = 0.46, *P* = 0.006, VIF = 1.07). Moreover, the timepoint the maximum velocity was achieved relative to the phase duration appeared to be predictive while preparing and actually attaching the tool to lift the cylinder out of the socket. While under early stimulation a later peak during the *Preparing* (*Preparing* TVP: $\beta$ = 0.30, *P* = 0.025, VIF = 2.02) and *Using* phase (*Using* TVP: $\beta$ = 0.34, *P* = 0.01, VIF = 1.99) increased the probability of classifying a trial as performed under verum stimulation, the opposite holds true for “late-stimulated” trials. Thus, an earlier velocity peak detection significantly influenced the classification as being performed under verum stimulation for both the *Preparing* (*Preparing* TVP: $\beta$ = −0.74, *P* $<0.001$, VIF = 2.03) and *Using* (*Using* TVP: $\beta$ = −0.53, *P* = 0.006, VIF = 1.93) phases.

### Model comparisons

**Figure 7:**
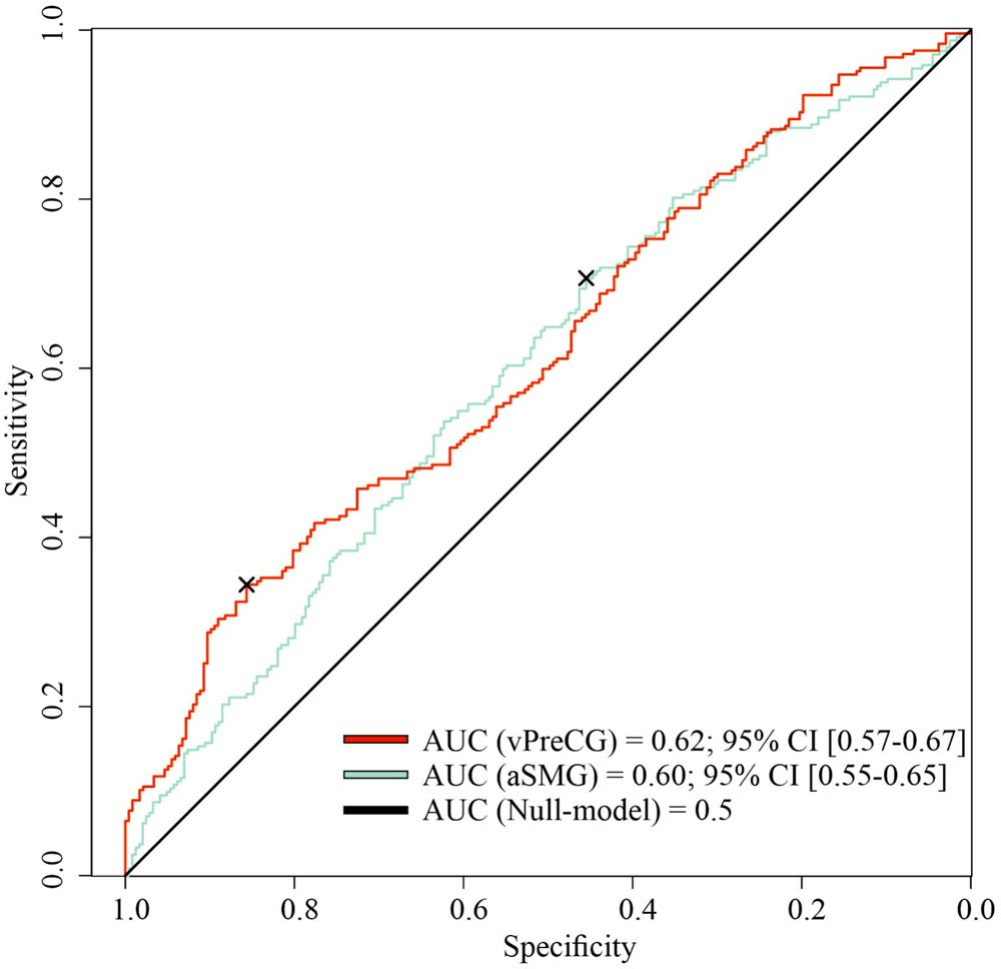
ROC curves for both logistic regression models. *Note.* Receiving operator characteristics (ROC) are indicated for both models as well as the Null-model without any predictor variable; the curves are further used to calculate the AUC; × indicate the best threshold points.

The receiving operator characteristic (ROC) curves for both logistic regression models are shown in Fig. 7. In an LRT, the performance of both models was separately compared against a Null-model that did not include any predictors. Both models outperformed the specified Null-model, with ${\chi}^2$(3) = 13.6, *P* = 0.003 for the aSMG and ${\chi}^2$(5) = 25.6, *P*  $<$ 0.001 for the vPreCG model. The calculated AUC revealed rates of 0.60, 95% CI [0.55, 0.65] for the aSMG model and 0.62, 95% CI [0.57, 0.67] for the vPreCG model.

## Discussion

The current study aimed to investigate the causal contribution of the aSMG and the vPreCG to the planning phases of tasks using novel tools that were assumed to require an individual’s mechanical problem solving ability in particular, by applying both verum and sham stimulation to healthy participants. The results indicated that neither the type nor the site of stimulation at given intensities significantly affected the overall selection and production scores. Due to the sham conditions being influenced by the stimulation site, two logistic regression models were created. Each model aimed to predict the type (verum/sham) of stimulation based on the kinematic data within its respective stimulation site. In the aSMG group during early stimulation, the prediction of the verum stimulation was associated with a longer trajectory traveled while reaching for the tool, as well as a reduced maximum velocity achieved when preparing to attach the tool to the cylinder. In the vPreCG group during late stimulation, kinematic parameters predicting verum stimulation included the longer time taken to initiate reaching for the tool, as well as earlier maximum velocities achieved while preparing the tool’s attachment and lifting the cylinder.

### No TMS effects on overall task performance

The stimulation protocol used in the current study did not significantly impact participants’ performance in either selecting the appropriate tool or in using the tool to lift the cylinder out of the socket. Although this point has not been explicitly discussed, previous studies involving healthy participants performing tool-related tasks also did not observe stimulation effects on overall task performance and the number of committed errors. For instance, the errors made in a finger-positioning task for object use by healthy participants after receiving stimulation to the SMG were not significantly negatively affected (Andres et al. 2017). In another study, healthy participants judged the manipulation associated with a specific tool or the context in which a tool is typically used while receiving stimulation to the left and right SMG (Pelgrims et al. 2011). Neither the location of the stimulation nor the task itself affected overall performance. However, exclusive stimulation of the left SMG led to prolonged verbal reaction times for tasks that required judgment about hand configuration (Pelgrims et al. 2011). Ishibashi et al. (2011) found that the errors made by healthy participants when rating the similarity of different tools’ functions or manipulations were not significantly influenced by the applied stimulation.

These previous studies aimed to apply a similar “virtual lesion” protocol as our current study. While the final goals of the applied tasks were achieved (successful task completion by providing the correct answer), the means of achieving these, mostly characterized by the time course of responses, were influenced by the given stimulation. These observations can, to some extent, be applied to the results presented here.

One possible explanation for these findings is that mechanisms were triggered to compensate for focal disruptions of the stimulated areas (aSMG or vPreCG). Compensation for these TMS-induced disturbances can occur within and across different networks (Hartwigsen 2018). Within-network compensation can be caused by another region in the same network that is not subjected to stimulation but supports the functionality of the inhibited area (Hartwigsen 2018). Alternatively, compensation driven by more dominant general cognitive processes may occur (Hartwigsen 2018). This type of compensation induces the activation of general cognitive processes, such as cognitive control, and has already been described following damage to a domain-specific language network (Brownsett et al. 2014). In our previous study, we noted that the network activated during the use of novel tools shared similarities with other networks reported to be active across a variety of different tasks (Seifert et al. 2025), such as the widely distributed fronto-parietal network (Duncan 2013). This overlapping activation may originate from the involvement of cognitive functions, supporting the ability to solve a mechanical problem, including spatial transformations, step-wise planning, and mental simulation processes, while maintaining focus on the intended goal (Seifert and Hermsdörfer 2025). These abilities are not exclusively important for successful task completion of the Novel Tool Test involving mechanical problem solving strategies but represent essential abilities for various other cognitively demanding tasks. The network activity, which supports more general cognitive functions, likely facilitated the final state to be successfully achieved, including the proper selection and usage of the novel tool, despite the given stimulation.

Another aspect to consider when interpreting the overall performance scores, which are unaffected by the given stimulation, refers to the scoring system itself. While this scheme enables a feasible and reliable evaluation of participants’ overall task performance, it may not be sensitive enough to detect subtle stimulation effects due to low resolution and ceiling effects when applied to healthy young participants. These smaller effects might be more easily identified by focusing on the kinematic profile of the hand movements.

### Kinematic parameters predict stimulation type within each stimulation site

Subtle kinematic characteristics were found to contribute classifying the stimulation type (verum/sham) within each site. To our knowledge, no study using TMS-induced “virtual lesions” has examined the predictive ability of kinematic characteristics in relation to stimulation factors, despite successful testing of their predictive value in other contexts (Kern et al. 2024). Although several kinematic differences between neurological patients and healthy controls were reported in past studies, which are used below to contextualize these findings, the interpretation of the identified kinematic characteristics remains largely exploratory.

According to the logistic regression model for the aSMG, under early stimulation conditions, the longer the path traveled while reaching for the most suitable tool, the more likely the trial was performed under verum stimulation. This finding may suggest indecisive behavior in selecting the most appropriate tool. Disruptions in early planning and decision-making processes may prevent the successful identification of the target tool, while mentally simulating which tool’s attachment is best suited to lift the cylinder and complete the task. Such behavior could be associated with a greater need for corrective movements while reaching and increments due to a less direct trajectory. A similar indecisive movement pattern was observed after stimulation of the right middle frontal gyrus, resulting in weakened decision-making for contralateral hand aiming movements (Gutierrez-Herrera et al. 2017). Another significant predictor includes the magnitude of the velocity profile while preparing the tool’s attachment. Maximum velocity is a kinematic measure reflecting movement control strategies (de los Reyes-Guzmán et al. 2014) and has been examined in patients with symptoms of apraxia, mostly revealing decreased maximum velocities (Hermsdörfer et al. 1996; Hermsdörfer et al. 2006; Mutha et al. 2010). While in our model, a decreased velocity magnitude predicts verum stimulation under early stimulation, an increased velocity magnitude predicts verum stimulation under late stimulation. Thus, the kinematic pattern found for early stimulation is similar to the one observed in ipsilateral movements of left brain-damaged patients (Hermsdörfer et al. 1996; Hermsdörfer et al. 2006; Mutha et al. 2010). During the *Preparing* phase, an analysis of the tool-cylinder characteristics is needed to determine the appropriate direction, orientation, and angle needed to attach the tool to the cylinder and to further solve the problem. A reduced maximum velocity may be related to challenges in building mental representations during early cognitive processing stages. This may lead to a slower, more controlled movement while bringing the tool toward the cylinder, allowing for a better matching of visual and material characteristics, as soon as the tool is closer to the cylinder (Hermsdörfer et al. 1996). This detailed processing of visual and structural features may be particular important abilities needed to solve the current task. In contrast, a higher maximum velocity increases the probability to predict verum stimulation under late stimulation. The increased maximum velocity may reflect the attempt to compensate for initial difficulties in mentally processing the relationship between the tool and the cylinder. This difficulty leads individuals to increase their maximum velocities as they move the tool toward the cylinder and prepare the attachment to complete the tasks successfully and on time.

The longer time taken by participants to start reaching during late vPreCG stimulation partially aligns with previous TMS study results showing prolonged reaction times in tool-related tasks (Ishibashi et al. 2011; Pelgrims et al. 2011). While these studies measured verbal or button responses to tool-related stimuli presented visually on a screen, the current study offers new insights into prolonged processes until the hand starts to move when healthy participants use real, tangible novel tools. The extended time beginning to reach may be attributed to small challenges in identifying the target tool, which may be due to slight interruptions in relating the tool-cylinder characteristics needed to achieve the final goal. In addition to this explanation, the predictive value of the phase duration in response to the starting signal may be due to the transience of the delivered pulses, exerting their strongest influence on kinematic parameters immediately after the pulses are released (Rice Cohen et al. 2009). Moreover, for late-stimulated trials, an earlier maximum velocity achieved during both the *Preparing* and the *Using* phase significantly increases the probability being performed under verum stimulation. These findings can be well contextualized within previous research, showing earlier velocity peaks and longer deceleration periods as participants approached a targeted tool in a cradle after stimulation of the left and right SMG (McDowell et al. 2018). Similarly, earlier peak velocity timings during grasping conditions when stimulating the aIPS were observed (Rice Cohen et al. 2009). This observation suggested that greater uncertainty during grasping led to a quick opening of the hand, followed by slower movement when approaching the object (Rice Cohen et al. 2009). Also, Rice et al. (2007) reported an earlier maximum velocity during grasping when stimulating the aIPS contralateral to the hand used, interpreted as a TMS-induced movement impairment, which could be compensated by allowing more time for the hand to reach the target object (Rice et al. 2007). The current study builds on these findings, with an earlier velocity peak reached under late verum stimulation of the vPreCG. The cognitive effort required during the *Preparing* phase may need an extended deceleration period as soon as the tool is moved toward the cylinder. A similar movement pattern is observed during the *Using* phase, which involves attaching the tool to the cylinder and further lifting the cylinder out of the socket. This may be a strategy to reduce the risk to fail during the final phase of task completion, requiring a controlled and slower lifting process.

In contrast, when velocity peaks occur later, they seem to be predictive of verum stimulation under early stimulation conditions. This pattern holds true for both the *Preparing* and *Using* phases. When interpreting this finding, one important consideration is the meaning and consequences of achieving a later velocity peak in the applied task, specifically while preparing and carrying out the tool-cylinder attachment. A later velocity peak may suggest impaired movement behavior, as it is followed by shorter deceleration phases and more hurried movements, even though more controlled and refined hand movements would probably be advantageous as soon as the tool approaches and is attached to the cylinder. Alternatively, the divergent characteristics of velocity peaks following early and late stimulation to the vPreCG may provide insights into the specific role of the vPreCG in this task. If the delayed velocity peaks observed under early stimulation conditions do not represent a clear negative behavioral consequence of vPreCG stimulation, these findings may suggest that the vPreCG is primarily involved in later cognitive processing stages. This time-dependent involvement may explain the different kinematic characteristics observed during early and late stimulation of this region. During late cognitive processing stages, the mental representations of how to best use the tool to achieve the goal, including the proper preparation and attachment, must be transformed into clear action plans. This process may require a well-elaborated analysis of the tool-cylinder relationship, as well as potential modifications to address circumstances that differ from the initial mental representation, ensuring that the final goal can still be achieved. This cognitive ability may be impaired if late cognitive processes, supported by the vPreCG, are disrupted.

While the current data do not permit a direct comparison of stimulation effects between the two regions involved in the left ventro-dorsal stream, (speculative) assumptions about the roles of the left aSMG and the vPreCG in this task can be made. The reported aSMG model may indicate that healthy participants subjected to aSMG stimulation experience slight challenges in reaching the appropriate tool, together with minor disturbances in simulating and creating mental representations of how to match the tool-cylinder characteristics when both are positioned at a distance from each other. These disturbances may particularly occur during early stimulation, resulting in longer reaching paths and slower, more controlled hand movement while preparing the tool’s attachment.

Although the vPreCG is assumed to form part of a motor network (Kantak et al. 2012), overall movement deficits such as reduced smoothness (less fluid movements) or decreased maximum velocities were not observed. However, trials performed under vPreCG stimulation tend to show a prolonged time starting to reach and an earlier velocity peak achievement when preparing and using the selected tool, particularly when later planning processes are disturbed. This finding may represent an individual’s strategy to compensate for disrupted late planning processes by decelerating the movement as the tool approaches the cylinder. It may indicate that the vPreCG may be rather involved in later cognitive processing stages, supporting an elaborated plan of how to exactly solve the apparent mechanical problem in the current situation.

### Limitations

One significant limitation of the current study is the kinematic differences observed in the sham conditions between the two stimulation sites. This difference allowed for accurate classification of stimulation sites by purely including trials performed under sham stimulation. This phenomenon may be attributed to methodological artifacts caused by the stimulation sites. While the sham coil delivers a low but distributed magnetic field, slight facial muscle twitches may be induced when stimulating areas that are located more frontal (vPreCG) compared to parietal (aSMG), which may have been distracting. Also, it needs to be considered that during frontal (vPreCG) compared to parietal (aSMG) stimulation, the coils may have been placed within the visual field, potentially influencing participants’ behavior.

Another limitation refers to the lack of stimulation effects on overall task performance. Although the explanation seems plausible that the overall task performance was unaffected by the given stimulation due to compensation by upregulating networks that support more general cognitive processes, there is no evidence for the actual inhibition of the target areas. Despite following a stimulation protocol designed to precisely target the selected areas, which included stimulation of task-related fMRI coordinates, individual brain scan acquisition for the neuronavigation procedure, and a real-time tracking of the coil’s placement, uncertainty remains regarding whether the applied stimulation achieved its intended effect. Moreover, the observation of lower selection scores when pulses were delivered early compared to late may indicate that participants were slightly distracted by the stimulation particularly when pulses were delivered closer to the opening of the goggles. Furthermore, while the two selected stimulation sites were derived from the findings of our previous fMRI study, we may have neglected the potential effects of other relevant regions within the activated network contributing to task performance.

### Improvement of model performance

The model selection procedure identified relevant kinematic parameters contributing to properly classify the stimulation type. Both models performed better than the specified Null-model; however, discriminatory ability could have been improved further. One way to achieve this improvement relates to the applied stimulation protocol, which induces the virtual lesion. A potential protocol modification mainly addresses two aspects: first, modifying the pulses delivered (eg triple pulses replaced by continuous theta-burst stimulation; Huang et al. 2005). Continuous theta-burst stimulation, however, would have impeded the discriminative analysis of earlier versus later stimulation times during the planning process. Second, following a more personalized target identification procedure, by purely stimulating individual task-related peak coordinates identified in the previous fMRI study, thus including only participants who also took part in the previous fMRI study (considering a sufficiently long break between the studies to further assume that mechanical problem solving represents the dominant cognitive process instead of recalling remembered tool-cylinder combinations). Another domain potentially improving model performance relates to the kinematic data gained from the specific task we applied. Past kinematic studies investigated repetitive movements, such as hammering (Hermsdörfer et al. 2013), bread slicing (Clark et al. 1994), or sawing (Hermsdörfer et al. 2006). These tasks probably lead to more movement cycles, resulting in kinematic characteristics that are less prone to errors or deviations, and a higher standardization of the executed movement. The specific task applied in the current study led to a relatively small number of trials performed, resulting in low statistical power. This, however, was needed as only a limited number of tool-cylinder combinations were available, and the study aimed to maintain the novel characteristic of the task, which makes it difficult to implement a higher repetition.

## Conclusion

The question of whether the two regions (aSMG/vPreCG) identified in our previous fMRI study are causally responsible to use novel tools cannot be entirely answered based on the results provided. While the overall performance is not impaired by the stimulation, some compensatory mechanisms seem to counteract the effects of the given stimulation. These are reflected in trials subjected to aSMG stimulation, showing rather longer trajectories when reaching toward the tool following early stimulation, and reduced velocity magnitudes, the closer the tool is moved to the cylinder. This may point to subtle challenges in overall mental representations of tool-cylinder characteristics, which are particularly relevant during initial processing stages. Trials performed under late verum stimulation to the vPreCG are rather characterized by prolonged time starting to move the hand, as well as longer deceleration periods when the tool is prepared and actually attached to the cylinder. These kinematic features under late stimulation may point toward subtle deficits that have to be compensated when a detailed tool-cylinder analysis about the structure, orientation, and material composition is disturbed. The findings may give insights into the time-dependent role of the involved brain regions. Future analyses could enhance these results, eg by effective connectivity analyses using the underlying fMRI data, to model the stimulus-processing stream from parietal to more frontal regions.

## Supplementary Material

Supplements_Effects_of_left_ventro-dorsal_stream_stimulation_on_novel_tool_use_bhag035
