# Supplementary material for: Effects of left ventro-dorsal stream stimulation on novel tool use
Source: Cereb Cortex. 2026 Apr 7;36(4):bhag035. doi: 10.1093/cercor/bhag035 (PMC13064860; doi:10.1093/cercor/bhag035)
Supplement: Supplements_Effects_of_left_ventro-dorsal_stream_stimulation_on_novel_tool_use_bhag035 [file supplements_effects_of_left_ventro-dorsal_stream_stimulation_on_novel_tool_use_bhag035.docx]

### Supplementary material

### Effects of left ventro-dorsal stream stimulation on novel tool use

### Clara Seifert^1^*, Philipp Gulde^1^, Thabea Kampe^1^, Afra Wohlschläger^2^, Joachim Hermsdörfer^1^

### ^1^Chair of Human Movement Science, Department Sport and Health Sciences, TUM School of Medicine and Health, Technical University of Munich, Munich, Germany

### ^2^TUM-NIC, Department of Neuroradiology, TUM School of Medicine and Health, Technical University of Munich, Munich, Germany

*Corresponding author: [clara.seifert@tum.de](mailto:clara.seifert@tum.de)

Chair of Human Movement Science, Department Sport and Health Sciences, TUM School of Medicine and Health, Technical University of Munich, Munich, Germany

Am Olympiacampus 11, 80809 München, Germany

#### Table S1: Full logistic regression model specification to predict the stimulation type in the aSMG stimulation group.

####

| Phase | Predictor | estimate | std.error | *p*.value |
| --- | --- | --- | --- | --- |
|  | Intercept | -0.05 | 0.19 | 0.78 |
|  | Timepoint of stimulation | 0.03 | 0.24 | 0.91 |
|  | Selection | 0.03 | 0.15 | 0.84 |
|  | Production | -0.06 | 0.15 | 0.70 |
| *Entire trial* | Trial duration | 21.45 | 15.86 | 0.18 |
| *Entire trial* | Relative activity | -0.85 | 0.58 | 0.14 |
| *Entire trial* | Number of velocity peaks per meter | 0.03 | 0.30 | 0.91 |
| *Go Cue* | Trial duration | -8.48 | 5.90 | 0.15 |
| *Reaching* | Trial duration | -9.63 | 7.10 | 0.17 |
| *Reaching* | Relative activity | 0.27 | 0.28 | 0.32 |
| *Reaching* | Pathlength | 0.50 | 0.32 | 0.11 |
| *Reaching* | Number of velocity peaks per meter | 0.04 | 0.20 | 0.86 |
| *Reaching* | Maximum velocity | 0.08 | 0.23 | 0.72 |
| *Reaching* | Timepoint of maximum velocity | 0.15 | 0.17 | 0.36 |
| *Preparing* | Trial duration | -9.12 | 7.21 | 0.21 |
| *Preparing* | Relative activity | 0.14 | 0.22 | 0.51 |
| *Preparing* | Pathlength | -0.60 | 0.45 | 0.18 |
| *Preparing* | Number of velocity peaks per meter | 0.04 | 0.20 | 0.84 |
| *Preparing* | Maximum velocity | -0.10 | 0.28 | 0.71 |
| *Preparing* | Timepoint of maximum velocity | 0.24 | 0.17 | 0.14 |
| *Using* | Trial duration | -16.51 | 12.08 | 0.17 |
| *Using* | Relative activity | 0.41 | 0.42 | 0.32 |
| *Using* | Pathlength | 0.34 | 0.40 | 0.40 |
| *Using* | Number of velocity peaks per meter | -0.01 | 0.20 | 0.97 |
| *Using* | Maximum velocity | -0.05 | 0.26 | 0.86 |
| *Using* | Timepoint of maximum velocity | -0.15 | 0.16 | 0.34 |
| *Entire trial* | Timepoint of stimulation × Trial duration | -17.61 | 16.68 | 0.29 |
| *Entire trial* | Timepoint of stimulation × Relative activity | 0.62 | 0.77 | 0.42 |
| *Entire trial* | Number of velocity peaks per meter | 0.29 | 0.41 | 0.48 |
| *Go Cue* | Timepoint of stimulation × Trial duration | 7.24 | 6.21 | 0.24 |
| *Reaching* | Timepoint of stimulation × Trial duration | 7.60 | 7.46 | 0.31 |
| *Reaching* | Timepoint of stimulation × Relative activity | -0.35 | 0.37 | 0.35 |
| *Reaching* | Timepoint of stimulation × Pathlength | -0.04 | 0.43 | 0.93 |
| *Reaching* | Timepoint of stimulation × Number of velocity peaks per meter | -0.19 | 0.28 | 0.49 |
| *Reaching* | Timepoint of stimulation × Maximum velocity | -0.19 | 0.33 | 0.56 |
| *Reaching* | Timepoint of stimulation × Timepoint of maximum velocity | -0.08 | 0.24 | 0.74 |
| *Preparing* | Timepoint of stimulation × Trial duration | 7.96 | 7.58 | 0.29 |
| *Preparing* | Timepoint of stimulation × Relative activity | -0.17 | 0.30 | 0.58 |
| *Preparing* | Timepoint of stimulation × Pathlength | -0.28 | 0.64 | 0.67 |
| *Preparing* | Timepoint of stimulation × Number of velocity peaks per meter | -0.08 | 0.28 | 0.78 |
| *Preparing* | Timepoint of stimulation × Maximum velocity | 0.89 | 0.41 | 0.03* |
| *Preparing* | Timepoint of stimulation × Timepoint of maximum velocity | -0.53 | 0.24 | 0.03* |
| *Using* | Timepoint of stimulation × Trial duration | 13.45 | 12.70 | 0.29 |
| *Using* | Timepoint of stimulation × Relative activity | -0.36 | 0.57 | 0.53 |
| *Using* | Timepoint of stimulation × Pathlength | -0.40 | 0.62 | 0.52 |
| *Using* | Timepoint of stimulation × Number of velocity peaks per meter | -0.11 | 0.30 | 0.71 |
| *Using* | Timepoint of stimulation × Maximum velocity | 0.23 | 0.43 | 0.59 |
| *Using* | Timepoint of stimulation × Timepoint of maximum velocity | -0.12 | 0.23 | 0.59 |
| *Note.* * *p*-value <.05 | | | | |

#### Table S2: Reduced logistic regression model specification to predict the stimulation type in the aSMG stimulation group, stepwise removal of predictors with VIF <5.

| Phase | Predictor | estimate | std.error | *p*.value | VIF |
| --- | --- | --- | --- | --- | --- |
|  | Intercept | 0.14 | 0.14 | 0.33 |  |
|  | Timepoint of stimulation (Late) | -0.18 | 0.19 | 0.34 | 1.07 |
|  | Selektion | 0.05 | 0.14 | 0.72 | 2.16 |
|  | Produktion | -0.08 | 0.15 | 0.57 | 2.35 |
| *Go Cue* | Trial duration | -0.09 | 0.20 | 0.66 | 3.99 |
| *Reaching* | Relative activity | 0.06 | 0.16 | 0.71 | 2.67 |
| *Reaching* | Pathlength | 0.43 | 0.19 | 0.02* | 3.42 |
| *Reaching* | Number of velocity peaks per meter | 0.06 | 0.16 | 0.70 | 2.67 |
| *Reaching* | Maximum velocity | 0.19 | 0.17 | 0.28 | 3.10 |
| *Reaching* | Timepoint of maximum velocity | 0.15 | 0.16 | 0.37 | 2.91 |
| *Preparing* | Trial duration | 0.02 | 0.16 | 0.90 | 2.75 |
| *Preparing* | Relative activity | -0.09 | 0.18 | 0.60 | 2.49 |
| *Preparing* | Number of velocity peaks per meter | 0.14 | 0.17 | 0.41 | 3.07 |
| *Preparing* | Maximum velocity | -0.36 | 0.19 | 0.06* | 3.57 |
| *Preparing* | Timepoint of maximum velocity | 0.21 | 0.16 | 0.18 | 2.62 |
| *Using* | Trial duration | -0.05 | 0.17 | 0.77 | 3.39 |
| *Using* | Relative activity | -0.14 | 0.18 | 0.43 | 3.36 |
| *Using* | Number of velocity peaks per meter | 0.02 | 0.14 | 0.88 | 2.23 |
| *Using* | Maximum velocity | 0.12 | 0.18 | 0.50 | 3.31 |
| *Using* | Timepoint of maximum velocity | -0.09 | 0.15 | 0.53 | 2.47 |
| *Go Cue* | Timepoint of stimulation × Trial duration | 0.33 | 0.24 | 0.17 | 3.92 |
| *Reaching* | Timepoint of stimulation × Relative activity | -0.07 | 0.23 | 0.76 | 2.84 |
| *Reaching* | Timepoint of stimulation × Pathlength | -0.24 | 0.24 | 0.32 | 3.32 |
| *Reaching* | Timepoint of stimulation × Number of velocity peaks per meter | -0.16 | 0.22 | 0.46 | 2.90 |
| *Reaching* | Timepoint of stimulation × Maximum velocity | -0.16 | 0.25 | 0.52 | 3.19 |
| *Reaching* | Timepoint of stimulation × Timepoint of maximum velocity | -0.01 | 0.23 | 0.98 | 2.96 |
| *Preparing* | Timepoint of stimulation × Trial duration | -0.08 | 0.24 | 0.74 | 2.51 |
| *Preparing* | Timepoint of stimulation × Relative activity | -0.17 | 0.25 | 0.49 | 2.58 |
| *Preparing* | Timepoint of stimulation × Number of velocity peaks per meter | 0.01 | 0.23 | 0.98 | 3.33 |
| *Preparing* | Timepoint of stimulation × Maximum velocity | 0.69 | 0.28 | 0.01* | 3.70 |
| *Preparing* | Timepoint of stimulation × Timepoint of maximum velocity | -0.47 | 0.23 | 0.04* | 2.65 |
| *Using* | Timepoint of stimulation × Trial duration | -0.04 | 0.24 | 0.88 | 3.35 |
| *Using* | Timepoint of stimulation × Relative activity | 0.12 | 0.25 | 0.63 | 3.52 |
| *Using* | Timepoint of stimulation × Number of velocity peaks per meter | 0.05 | 0.21 | 0.82 | 2.40 |
| *Using* | Timepoint of stimulation × Maximum velocity | -0.09 | 0.26 | 0.74 | 3.11 |
| *Using* | Timepoint of stimulation × Timepoint of maximum velocity | -0.17 | 0.21 | 0.41 | 2.49 |
| *Note.* * *p*-value <.10, ** *p*-value <.005. | | | | | |

#### Table S3: Final logistic regression model specification to predict the stimulation type in the aSMG stimulation group after removal of non-significant predictors.

| Phase | Predictor | estimate | std.error | *p*.value | VIF |
| --- | --- | --- | --- | --- | --- |
|  | Intercept | 0.02 | 0.09 | 0.860 |  |
| *Reaching* | Pathlength | 0.281 | 0.100 | 0.005* | 1.07 |
| *Preparing* | Maximum velocity | -0.28 | 0.131 | 0.032* | 1.99 |
| *Preparing* | Timepoint of stimulation (late) × Maximum velocity | 0.46 | 0.189 | 0.015* | 1.89 |
| *Note*. **p-*value <.05 | | | | | |

Table S4: Full logistic regression model specification to predict the stimulation type in the vPreCG stimulation group.

| Phase | Predictor | estimate | std.error | *p*.value |
| --- | --- | --- | --- | --- |
|  | Intercept | 0.31 | 0.29 | 0.29 |
|  | Timepoint of stimulation | -0.40 | 0.37 | 0.29 |
|  | Selection | 0.00 | 0.12 | 0.98 |
|  | Production | -0.07 | 0.13 | 0.62 |
| *Entire trial* | Trial duration | 19.05 | 28.89 | 0.51 |
| *Entire trial* | Relative activity | -0.29 | 0.28 | 0.30 |
| *Entire trial* | Number of velocity peaks per meter | -0.15 | 0.32 | 0.63 |
| *Go Cue* | Trial duration | -6.00 | 8.88 | 0.50 |
| *Reaching* | Trial duration | -8.26 | 11.92 | 0.49 |
| *Reaching* | Relative activity | -0.53 | 0.33 | 0.11 |
| *Reaching* | Pathlength | 0.39 | 0.36 | 0.28 |
| *Reaching* | Number of velocity peaks per meter | 0.16 | 0.21 | 0.43 |
| *Reaching* | Maximum velocity | 0.20 | 0.25 | 0.41 |
| *Reaching* | Timepoint of maximum velocity | -0.03 | 0.16 | 0.86 |
| *Preparing* | Trial duration | -7.24 | 11.10 | 0.51 |
| *Preparing* | Relative activity | 0.19 | 0.21 | 0.37 |
| *Preparing* | Pathlength | -0.42 | 0.34 | 0.22 |
| *Preparing* | Number of velocity peaks per meter | 0.34 | 0.24 | 0.16 |
| *Preparing* | Maximum velocity | 0.02 | 0.25 | 0.93 |
| *Preparing* | Timepoint of maximum velocity | 0.56 | 0.19 | 0.00** |
| *Using* | Trial duration | -15.29 | 22.59 | 0.50 |
| *Using* | Relative activity | -0.09 | 0.25 | 0.73 |
| *Using* | Pathlength | 0.38 | 0.58 | 0.51 |
| *Using* | Number of velocity peaks per meter | 0.20 | 0.23 | 0.39 |
| *Using* | Maximum velocity | -0.05 | 0.44 | 0.91 |
| *Using* | Timepoint of maximum velocity | 0.37 | 0.16 | 0.02* |
| *Entire trial* | Timepoint of stimulation × Trial duration | -37.25 | 36.89 | 0.31 |
| *Entire trial* | Timepoint of stimulation × Relative activity | -0.60 | 0.47 | 0.20 |
| *Entire trial* | Timepoint of stimulation × Number of velocity peaks per meter | 0.14 | 0.45 | 0.76 |
| *Go Cue* | Timepoint of stimulation × Trial duration | 11.69 | 11.35 | 0.30 |
| *Reaching* | Timepoint of stimulation × Trial duration | 16.87 | 15.22 | 0.27 |
| *Reaching* | Timepoint of stimulation × Relative activity | 0.97 | 0.38 | 0.01* |
| *Reaching* | Timepoint of stimulation × Pathlength | -0.95 | 0.52 | 0.07 |
| *Reaching* | Timepoint of stimulation × Number of velocity peaks per meter | -0.47 | 0.29 | 0.11 |
| *Reaching* | Timepoint of stimulation × Maximum velocity | -0.10 | 0.34 | 0.78 |
| *Reaching* | Timepoint of stimulation × Timepoint of maximum velocity | 0.14 | 0.22 | 0.54 |
| *Preparing* | Timepoint of stimulation × Trial duration | 13.74 | 14.15 | 0.33 |
| *Preparing* | Timepoint of stimulation × Relative activity | -0.11 | 0.31 | 0.72 |
| *Preparing* | Timepoint of stimulation × Pathlength | 1.11 | 0.56 | 0.05 |
| *Preparing* | Timepoint of stimulation × Number of velocity peaks per meter | -0.07 | 0.33 | 0.84 |
| *Preparing* | Timepoint of stimulation × Maximum velocity | -0.23 | 0.43 | 0.59 |
| *Preparing* | Timepoint of stimulation × Timepoint of maximum velocity | -1.06 | 0.26 | 0.00** |
| *Using* | Timepoint of stimulation × Trial duration | 29.63 | 28.86 | 0.30 |
| *Using* | Timepoint of stimulation × Relative activity | 0.80 | 0.43 | 0.06 |
| *Using* | Timepoint of stimulation × Pathlength | -0.15 | 0.77 | 0.84 |
| *Using* | Timepoint of stimulation × Number of velocity peaks per meter | -0.16 | 0.32 | 0.62 |
| *Using* | Timepoint of stimulation × Maximum velocity | -0.17 | 0.57 | 0.76 |
| *Using* | Timepoint of stimulation × Timepoint of maximum velocity | -0.64 | 0.23 | 0.01* |
| *Note.* * *p*-value <.05, ** *p*-value <.001 | | | | |

#### Table S5: Reduced logistic regression model specification to predict the stimulation type in the vPreCG stimulation group, stepwise removal of predictors with VIF <5.

| Phase | Predictor | estimate | std.error | *p*.value | VIF |
| --- | --- | --- | --- | --- | --- |
|  | Intercept | 0.15 | 0.14 | 0.29 |  |
|  | Timepoint of stimulation | -0.09 | 0.20 | 0.64 | 1.09 |
|  | Selection | 0.04 | 0.12 | 0.72 | 1.45 |
|  | Production | -0.08 | 0.13 | 0.52 | 1.64 |
| *Entire trial* | Relative activity | -0.44 | 0.21 | 0.04** | 4.32 |
| *Go Cue* | Trial duration | -0.20 | 0.16 | 0.20 | 2.62 |
| *Reaching* | Trial duration | 0.23 | 0.14 | 0.09* | 1.84 |
| *Reaching* | Relative activity | -0.19 | 0.28 | 0.50 | 1.26 |
| *Reaching* | Number of velocity peaks per meter | -0.02 | 0.17 | 0.92 | 2.84 |
| *Reaching* | Maximum velocity | 0.35 | 0.18 | 0.06* | 3.52 |
| *Reaching* | Timepoint of maximum velocity | -0.08 | 0.15 | 0.58 | 2.28 |
| *Preparing* | Relative activity | 0.22 | 0.16 | 0.16 | 2.51 |
| *Preparing* | Pathlength | -0.04 | 0.14 | 0.75 | 1.94 |
| *Preparing* | Number of velocity peaks per meter | 0.24 | 0.19 | 0.19 | 3.59 |
| *Preparing* | Maximum velocity | -0.16 | 0.20 | 0.42 | 4.48 |
| *Preparing* | Timepoint of maximum velocity | 0.45 | 0.16 | 0.01*** | 2.74 |
| *Using* | Trial duration | -0.24 | 0.17 | 0.16 | 3.15 |
| *Using* | Relative activity | 0.06 | 0.21 | 0.79 | 4.78 |
| *Using* | Number of velocity peaks per meter | 0.06 | 0.16 | 0.70 | 2.74 |
| *Using* | Maximum velocity | 0.20 | 0.20 | 0.34 | 4.56 |
| *Using* | Timepoint of maximum velocity | 0.34 | 0.16 | 0.03** | 2.52 |
| *Go Cue* | Timepoint of stimulation × Trial duration | 0.50 | 0.24 | 0.04** | 2.04 |
| *Reaching* | Timepoint of stimulation × Relative activity | 0.16 | 0.14 | 0.26 | 1.60 |
| *Reaching* | Timepoint of stimulation × Number of velocity peaks per meter | -0.12 | 0.22 | 0.60 | 2.54 |
| *Reaching* | Timepoint of stimulation × Maximum velocity | -0.51 | 0.24 | 0.04** | 3.50 |
| *Reaching* | Timepoint of stimulation × Timepoint of maximum velocity | 0.15 | 0.21 | 0.48 | 2.40 |
| *Preparing* | Timepoint of stimulation × Relative activity | -0.11 | 0.23 | 0.63 | 2.43 |
| *Preparing* | Timepoint of stimulation × Number of velocity peaks per meter | -0.05 | 0.26 | 0.84 | 3.84 |
| *Preparing* | Timepoint of stimulation × Maximum velocity | 0.41 | 0.28 | 0.15 | 3.94 |
| *Preparing* | Timepoint of stimulation × Timepoint of maximum velocity | -0.91 | 0.23 | 0.00*** | 2.65 |
| *Using* | Timepoint of stimulation × Trial duration | 0.39 | 0.25 | 0.12 | 3.04 |
| *Using* | Timepoint of stimulation × Relative activity | 0.36 | 0.25 | 0.15 | 2.78 |
| *Using* | Timepoint of stimulation × Number of velocity peaks per meter | -0.05 | 0.22 | 0.83 | 2.58 |
| *Using* | Timepoint of stimulation × Maximum velocity | -0.27 | 0.27 | 0.31 | 4.41 |
| *Using* | Timepoint of stimulation × Timepoint of maximum velocity | -0.59 | 0.22 | 0.01** | 2.49 |
| *Note. * p*-value <.10, **** *p*-value <.05, *** *p*-value <.001 | | | | | |

Table S6: Final logistic regression model specification to predict the stimulation type in the vPreCG stimulation group after removal of non-significant predictors.

| Phase | Predictor | estimate | std.error | *p*.value | VIF |
| --- | --- | --- | --- | --- | --- |
|  | Intercept | 0.33 | 0.09 | 0.731 |  |
| *Preparing* | Timepoint of maximum velocity | 0.30 | 0.14 | 0.025* | 2.02 |
| *Using* | Timepoint of maximum velocity | 0.34 | 0.13 | 0.01* | 1.99 |
| *Go Cue* | Trial duration | 0.46 | 0.17 | 0.006* | 1.07 |
| *Preparing* | Timepoint of stimulation (late) × Timepoint of maximum velocity | -0.74 | 0.19 | <.001** | 2.03 |
| *Using* | Timepoint of stimulation (late) × Timepoint of maximum | -0.53 | 0.19 | 0.006* | 1.93 |
| *Note*. **p-*value <.05, ** *p-*value <.001. | | | | | |

Figure S1: Overview of the different phases.


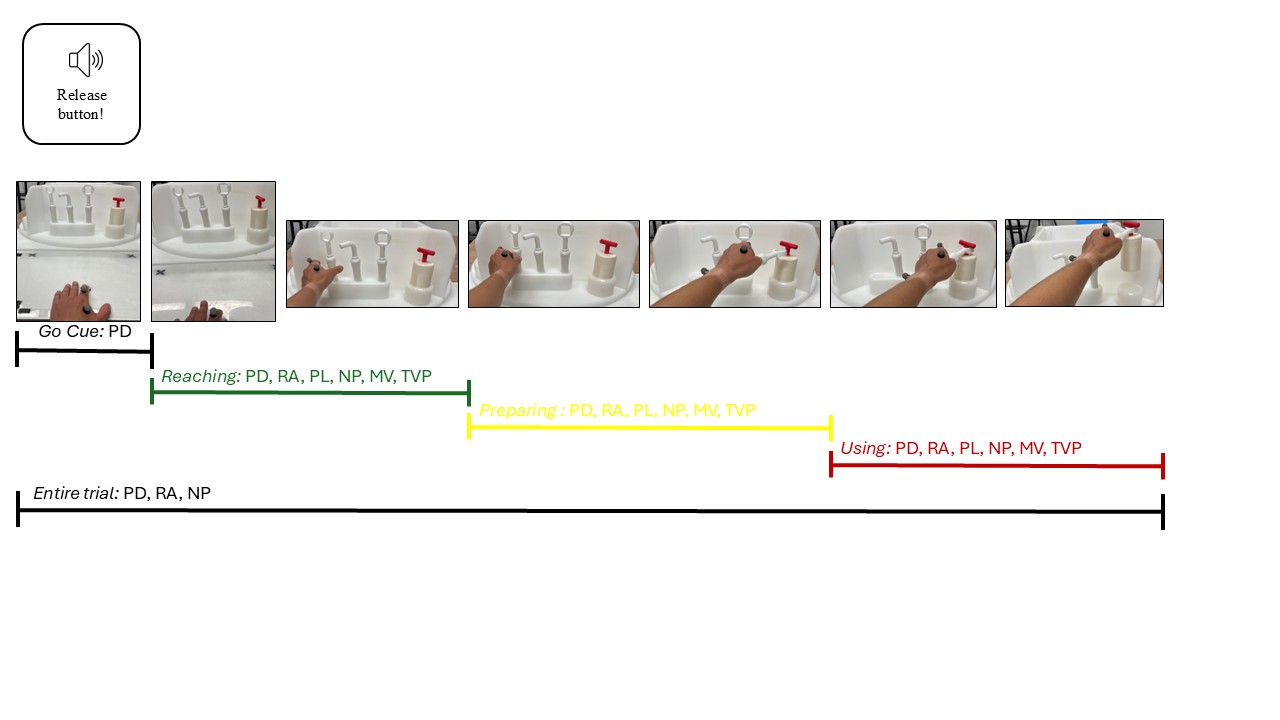


Figure S1. Visual representation of how the different phases were defined. The Go Cue phase covers the period from the start signal until the hand begins to move; the Reaching phase spans from the first movement of the hand to reaching the tool; the Preparing phase includes the time from grasping the tool to moving it toward the cylinder; the Using phase starts when the tool is attached to the cylinder and continues until the cylinder is lifted out of the socket. Various kinematic parameters were calculated for each phase: PD = phase duration, RA = relative activity, PL = path length, NP = number of velocity peaks per meter, MV = maximum velocity, TVP = timepoint of maximum velocity.

Figure S2: MRI compatible material from the Novel Tool Test.


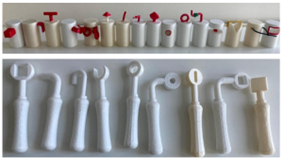


Figure S2. The upper part represents all available cylinders, including two cylinders for familiarization. The lower part represents all available novel tools with similar handles but different attachments.

Figure S3: aSMG Correlation matrix.
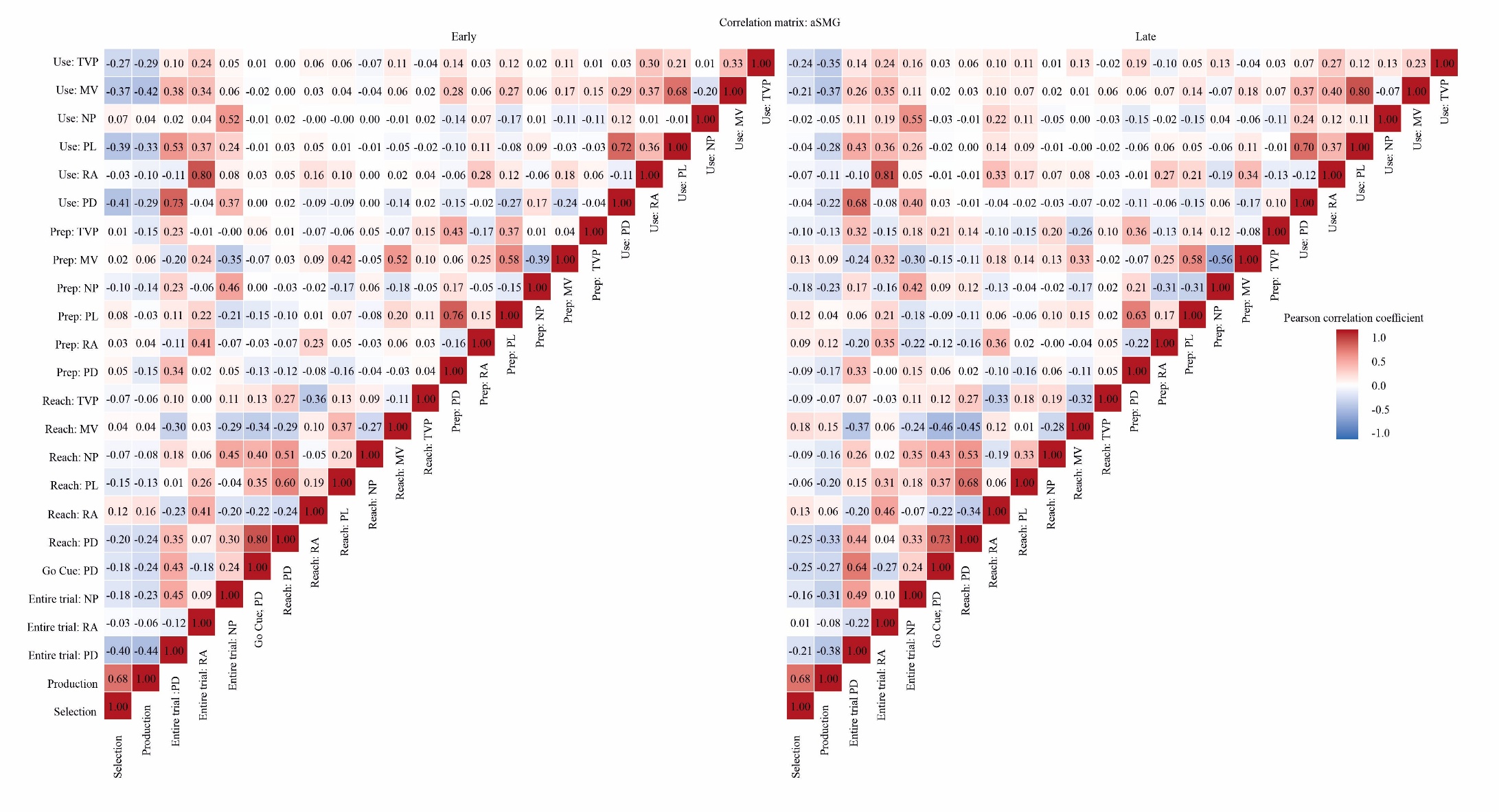


Figure S3. Correlation matrices among predictors for different phases (Use = using; Prep = preparing; Reach = reaching, Go Cue; Entire trial); kinematic parameters include TVP = timepoint of velocity peak, MV = maximum velocity, PL = pathlength, RA = relative activity, NP = number of velocity peaks, PD = phase duration); matrices are depicted separately for early and late stimulation for the aSMG group.

Figure S4: vPreCG Correlation matrix.
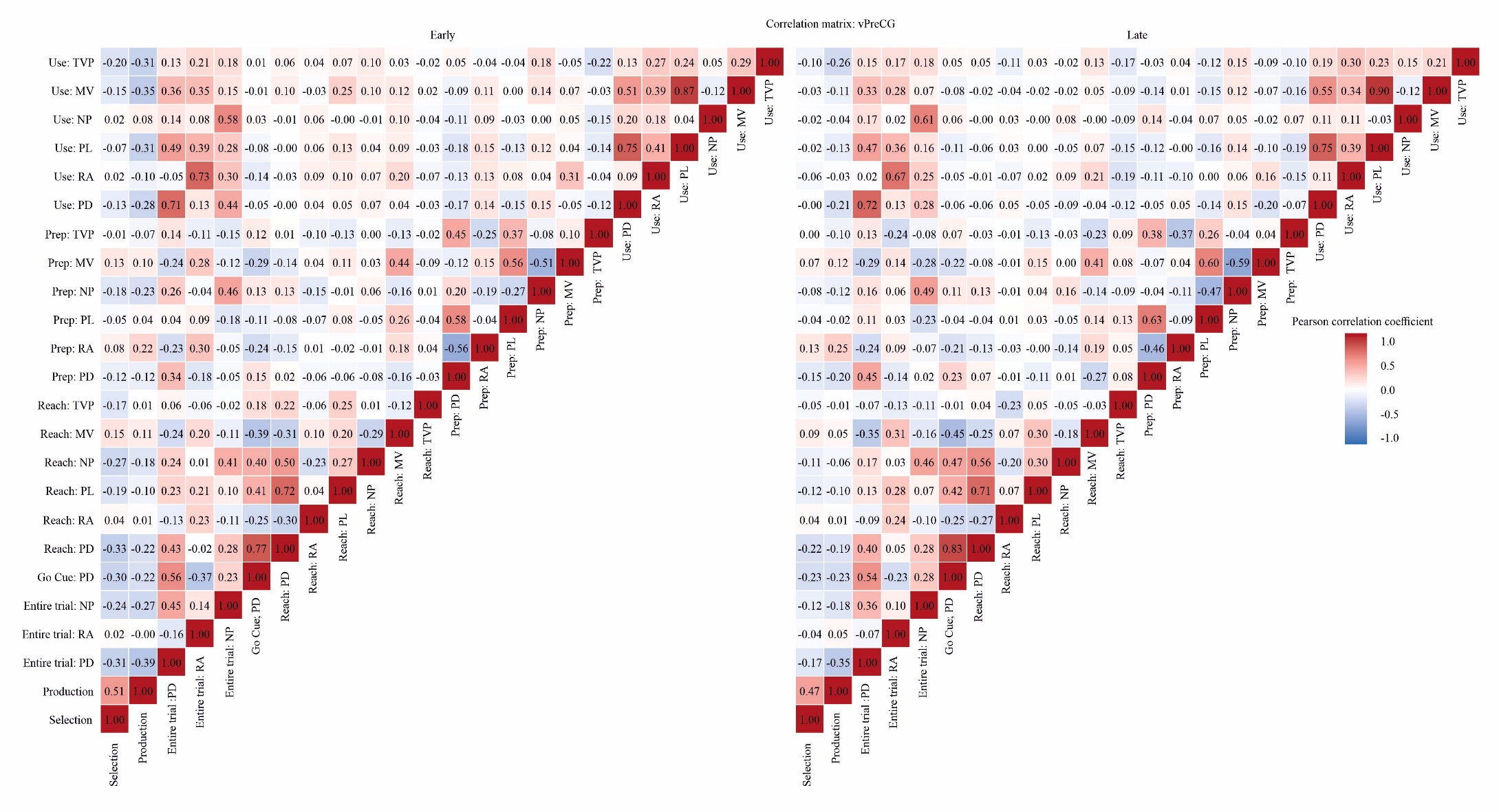


Figure S4. Correlation matrices among predictors for different phases (Use = using; Prep = preparing; Reach = reaching, Go Cue; Entire trial); kinematic parameters include TVP = timepoint of velocity peak, MV = maximum velocity, PL = pathlength, RA = relative activity, NP = number of velocity peaks, PD = phase duration); matrices are depicted separately for early and late stimulation for the vPreCG group.

Table S7: individual peak coordinates and behavioral outcomes for participants who took part in both (fMRI and TMS) studies.

| Individual peak coordinate (MNI space) | Averaged selection scores | Averaged production scores |
| --- | --- | --- |
| -46 \| 2\| 36 | 0.89 | 2.642 |
| -44\|4\|24 | 0.821 | 2.357 |
| -42\|8\|30 | 0.852 | 2.74 |
| -48\|12\|48 | 0.875 | 2.62 |
| *Note.* Selection scores varied from 0-1; Production scores varied from 0-3. | | |
